# *FUS*-linked essential tremor associated with motor dysfunction in *Drosophila*

**DOI:** 10.1007/s00439-016-1709-z

**Published:** 2016-07-09

**Authors:** Murni Tio, Rujing Wen, Yih Lin Lim, Huashan Wang, Shuo-Chien Ling, Yi Zhao, Eng-King Tan

**Affiliations:** 1 National Neuroscience Institute, 20 College Road, The Academia, Level 9 (Discovery Tower), Singapore, 169856 Singapore; 2 Yong Loo Lin School of Medicine, National University of Singapore, 12 Science Drive 2, Singapore, 117549 Singapore; 3 Department of Clinical Research, 20 College Road, The Academia, Level 9 (Discovery Tower), Singapore, 169856 Singapore; 4 Department of Neurology, Singapore General Hospital, National Neuroscience Institute, Outram Road, Singapore, 169608 Singapore; 5 Duke-NUS Graduate Medical School, 8 College Road, Singapore, 169857 Singapore

## Abstract

Essential tremor (ET) is one of the most common adult-onset neurological disorders which produce motor and non-motor symptoms. To date, there are no gold standard pathological hallmarks of ET, and despite a strong genetic contribution toward ET development, only a few pathogenic mutations have been identified. Recently, a pathogenic *FUS*-*Q290X* mutation has been reported in a large ET-affected family; however, the pathophysiologic mechanism underlying FUS-linked ET is unknown. Here, we generated transgenic *Drosophila* expressing *hFUS*-*WT* and *hFUS*-*Q290X* and targeted their expression in different tissues. We found that the targeted expression of *hFUS*-*Q290X* in the dopaminergic and the serotonergic neurons did not cause obvious neuronal degeneration, but it resulted in motor dysfunction which was accompanied by impairment in the GABAergic pathway. The involvement of the GABAergic pathway was supported by rescue of motor symptoms with gabapentin. Interestingly, we observed gender specific downregulation of *GABA*-*R* and *NMDA*-*R* expression and reduction in serotonin level. Overexpression of *hFUS*-*Q290X* also caused an increase in longevity and this was accompanied by downregulation of the IIS/TOR signalling pathway. Our in vivo studies of the *hFUS*-*Q290X* mutation in *Drosophila* link motor dysfunction to impairment in the GABAergic pathway. Our findings would facilitate further efforts in unravelling the pathophysiology of ET.

## Introduction

Essential tremor (ET) is one of the most common adult-onset, age-progressive neurological disorders, affecting about 0.9 % of world population (Louis and Ferreira 2010). Despite its high prevalence, the exact pathophysiology of ET remains to be elucidated. ET is a heterogeneous disorder involving both motor (such as tremor) and non-motor (such as cognitive, psychiatric, dementia and sensory) symptoms (Chandran and Pal 2012). Unlike in neurodegenerative condition such as PD where there is a progressive loss of dopaminergic neurons (Benazzouz et al. 2014), no obvious neuronal loss has been reproducibly observed for ET. Neuropathological evidence suggests abnormalities in the GABAergic Purkinje cells of the cerebellum and their surrounding neuronal populations which likely lead to defects in the cerebellar cortical circuits and output (Louis 2016). Higher mortality rates have been reported in the cases of late-onset ET (Louis et al. 2007), but increased longevity has also been associated with early onset ET and observed in ET patients and their relatives (Jankovic et al. 1995).

Gene seems to play a major role on the development of ET (Jimenez-Jimenez et al. 2013), although the exact cause and effect relationship is unclear. This is due to limited discoveries of pathogenic ET gene mutations and lack of in vivo ET models. Pathogenic risk variants have been reported for *TENM4* (teneurin transmembrane protein 4), and studies of this gene in zebra fish suggested its role on oligodendrocyte differentiation and myelination of CNS axons (Hor et al. 2015). A pathogenic mutation (*Q290X*) has also been recently identified for the *FUS/TLS* (fused in sarcoma/translated in liposarcoma) gene through exome sequencing in a large ET family from Quebec (Merner et al. 2012). FUS is an RNA/DNA binding protein, and it is involved in the regulation of transcription, RNA splicing, and translational control (Prasad et al. 1994; Belly et al. 2005). *Q290X* is a nonsense mutation caused by a single-base-pair substitution which results in the truncation of the FUS protein at amino acid 290 in its nuclear export signal motif (Merner et al. 2012). Mutations in other domains of the FUS protein are known to cause amyotrophic lateral sclerosis (ALS) and frontotemporal dementia (FTD) (Vance et al. 2009; Chen et al. 2011).

Here, we performed targeted expression analysis (Brand and Perrimon 1993) of *hFUS*-*Q290X* in *Drosophila* and characterised its phenotype. In addition, we examined the involvement of the GABAergic system, the serotonergic system, and the IIS/TOR signalling pathway, and conducted therapeutic challenges.

## Materials and methods

### *Drosophila* strains and generation of transgenic flies

Flies were raised on standard corn meal containing food and genetic crosses were performed at 25 °C. GAL4 lines (*GMR*-*GAL4*, *ple*-*GAL4*, *Ddc*-*GAL4* and *OK371*-*GAL4*) and RNAi lines to *dFUS* (stock numbers 32990 and 34839) used were obtained from Bloomington *Drosophila* Stock Centre (BDSC). For generation of the wild-type transformation construct, wild-type *hFUS* cDNA was excised from the pCMV-FUS plasmid DNA (from Origene) using restriction enzymes XhoI and XbaI and subcloned into the XhoI and XbaI digested pUAST-attB transformation vector. The *Q290X* mutation was generated using the Quick-Change II Site-Directed Mutagensis Kit (Stratagene) following the manufacturer’s instructions, with the pUAST-attB-wild-type hFUS as the DNA template. All plasmid constructs which had been subcloned into the transformation vector were verified by sequencing before sent for transgenesis at Best Gene Inc. To avoid possible contribution of second site mutation which might have arisen during transgenesis, the transgenic lines were backcrossed into yw (from Bloomington) for six generations.

### Western blotting

Proteins were extracted from head homogenates of approximately 30–50 flies of the respective genotypes. Adult flies were exposed to liquid nitrogen followed by shaking to separate the heads from the bodies. Heads were collected and homogenised in M-PER Mammalian Protein Extraction Buffer (Thermo Scientific) in the presence of protease inhibitor (Roche). Cell debris was removed by centrifugation, and protein concentrations were determined by BCA protein assay (Thermo Scientific). 50–150 µg of total proteins of each samples were denatured by heating to 95 °C and loaded on SDS–PAGE gels. After electrophoresis, proteins were transferred to nitrocellulose paper, blocked with 1 × PBST + 5 % non-fat milk and incubated in primary antibody overnight at 4 °C. This was followed by incubation in secondary antibody. Protein expression was detected by chemiluminescence (Thermo Scientific). Primary antibodies used were rabbit anti-FUS (from Bethyl Labs, cat. #A300-302A, 1:1000 dilution), rabbit anti-N-terminal hFUS (LSC, unpublished result, 1:1000), mouse anti-serotonin (Thermo Scientific, 1:40 dilution), and mouse anti-tubulin (Sigma, 1:10,000 dilution). Secondary antibodies used were HRP-conjugated goat anti-mouse/rabbit (Santa Cruz Biotechnology, Inc., 1:4000 dilution).

### Immunohistochemistry

Brain fixation and antibody staining were carried out according to the standard protocol. Briefly, adult brains were dissected in phosphate buffered saline (PBS) then fixed with 4 % formaldehyde followed by a few washes in PBT (PBS + 0.1 % Triton X-100). After blocking with 3 % BSA (in PBT), brain samples were incubated in primary antibody (rabbit anti-tyrosine hydroxylase, Sigma, 1:1000 dilution) overnight at 4 °C with rotation. After removal of primary antibodies, samples were washed with PBT followed by incubation in secondary antibody (Cy3-conjugated goat anti-rabbit, Jackson Immunoresearch, 1:500). After washes, samples were incubated with Vectashield and analysed by Carl Zeiss Upright Confocal Microscope.

### Light microscopy of adult eyes and wings

For adult eye microscopy, heads were removed and glued to masking tapes, and then, photos were captured by a digital camera through eye piece of a scanning microscope (Olympus). Photography of wing phenotypes was carried out similarly by placing flies on CO_2_ gas pad on their backs.

### Longevity and climbing assays

Flies were crossed at 25 °C on standard corn meal containing food, and at day 1 post-eclosion, males and females were separated. Adult flies were aged at 25 °C. Life span was scored at a density of 20 flies per vials. Flies were transferred to fresh vials thrice a week, followed by scoring of dead flies at every transfer. Dead flies were confirmed under microscope to assure that they had stopped moving completely. Locomotion was analysed using negative geotaxis assay where approximately 20 flies were left to acclimatise to 20-cm height in 1 min. The percentage of flies that climbed passed the 20-cm height was recorded. For drug treatment, female flies were aged to 10 days, then exposed to drug and subjected to negative geotaxis assay. Gabapentin (Sigma–Aldrich) was mixed in the food at the concentration of 0.1 mg/ml according to the published literature (Reynolds et al. 2004).

### RT-qPCR

For qPCR, approximately 150–200 fly heads were homogenised on ice. Total RNAs were extracted using the RNeasy mini kit (Qiagen) according to manufacturer’s instructions. Conversion to cDNA was done using High-Capacity Reverse Transcription kit (Applied Biosystems), with 1 µg each of RNA template. qPCR was carried out using GoTaq^®^ qPCR Master Mix (Promega) with the 7500 Fast Real Time System (Applied Biosystems). Primer sequences used to detect *Drosophila* melanogaster mRNA expressions were as follows: *GABA*_*A*_-*R* (Fwd: 5′-GCGAAATTCAGTTCGTGCGT-3′, Rev: 5′-ACAACGATCAGTCCAGAGGG-3′), *GABA*_*B*_-*R* (Fwd: 5′-CAACGACAGCGAGTGTGAG-3′, Rev: 5′-GATGGGTGCGGAATAGAGTG-3′), *NMDA*-*R1* (Fwd: 5′-GTGGAGCTCTCCAACATGTATC-3′, Rev: 5′-AATCCCAGATGAAGGCCATAAG-3′), *NMDA*-*R2* (Fwd: 5′-TCCTACAGCGAGTACGTCTAA-3′, Rev: 5′-CTTTCGCTATCTCGTCCCTAAC-3′), *4E*-*BP* (Fwd: 5′-TCAGCTAAGATGTCCGCTTC-3′, Rev: 5′-AGATAAGTTTGGTGCCTCCA-3′ and *atub84b* (Fwd: 5′-GATCGTGTCCTCGATTACCGC-3′, Rev: 5′-GGGAAGTGAATACGTGGGTAGG-3′). Primer sequences used to detect expression of *hFUS* from transgenes were Fwd: 5′-ACGGACACTTCAGGCTATGG-3′ and Rev: 5′-TACCGTAACTTCCCGAGGTG-3′. Calculation of relative gene expression from RT-qPCR experiments was performed using the 2(−Delta Delta C(T)) method (Livak and Schmittgen 2001).

### Statistical analysis

For all experiments, data from at least two to three biological replicates were obtained. For each biological replicate, three rounds of technical replicates were conducted. In this case, biological replicates involved samples obtained from separate batches of genetic crosses which were conducted repeatedly. Technical replicates involved samples from same batches of genetic crosses. Negative geotaxis assay was analysed by Pearson Chi-square (d*f* = 1) on data obtained from three biological replicates. RT-qPCR analysis of *FUS* mRNA levels was tested using one-way ANOVA with Bonferroni post-test for multiple comparisons on data obtained from two biological replicates. RT-qPCR analysis of *GABA*-*R*, *NMDA*-*R,* and *4E*-*BP* expression levels was tested using one sample Student’s *t* test (95 % confidence interval) on data obtained from three biological replicates. Protein band quantification on Western Blot (quantified using Image J program) was tested using one sample Student’s *t* test (95 % confidence interval) on data obtained from three and five biological replicates (for Western Blot results of FUS protein and for Serotonin levels, respectively). Longevity assay was analysed using Kaplan–Meier analysis with Cox regression (95 % confidence interval) on data obtained from five biological replicates. Software used to perform statistical analysis was IBM-SPSS Statistical 21. Significant differences were defined as *P* < 0.05.

## Results

### Phenotypic characterization of *hFUS*-*Q290X* in *Drosophila*

**Fig. 1:**
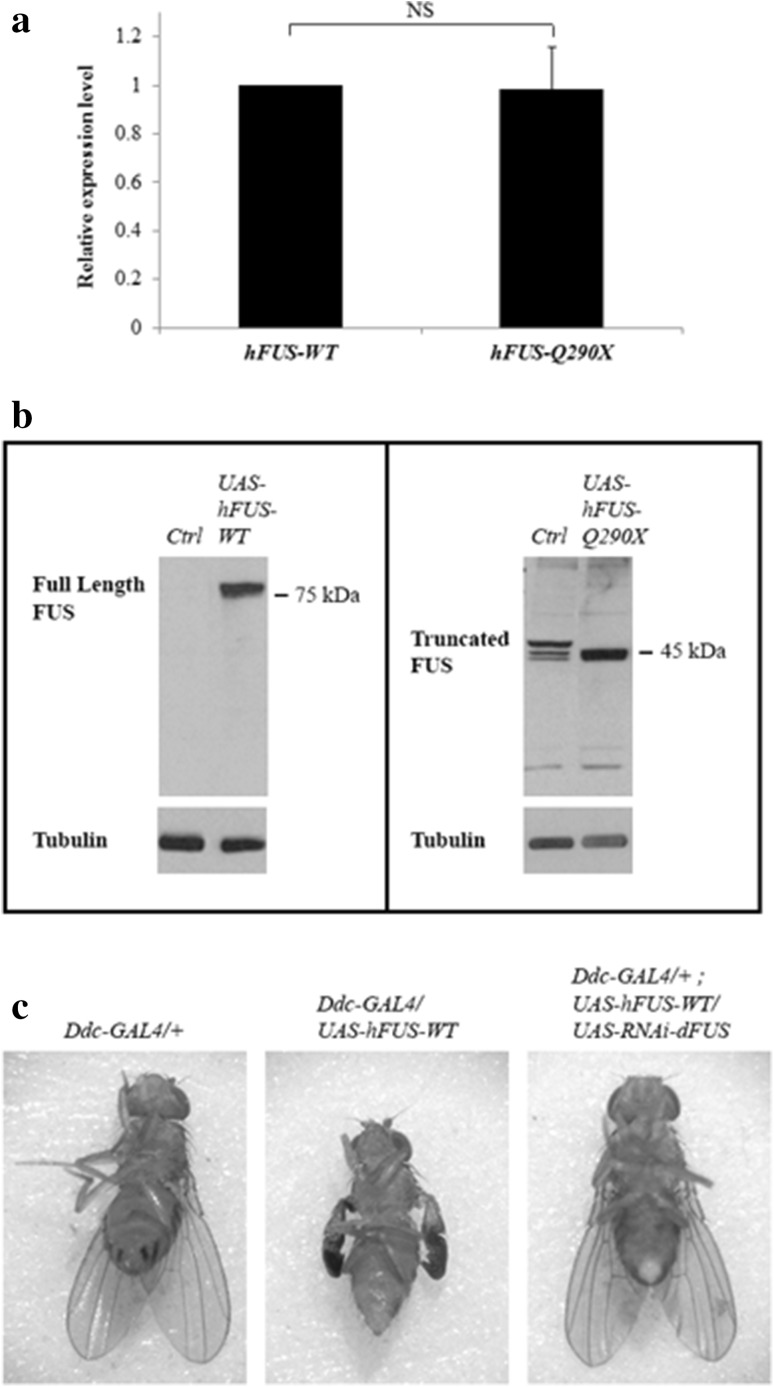
Expression from the *hFUS* transgenes and the ability of knock down of endogenous *dFUS* to rescue wing defect caused by ectopic expression of *hFUS*-*WT*. **a** RT-qPCR showing mRNA expression from transgenic lines carrying *UAS*-*hFUS*-*WT* and *UAS*-*hFUS*-*Q290X* (after 15 days of aging), driven by *Ddc*-*GAL4*. The relative expression levels of the two transgenes are shown. Note that both transgenic lines have similar expression levels. Statistics used is one-way ANOVA with Bonferroni post-test for multiple comparisons. **b** Western Blot analysis showing protein expression of full-length hFUS-WT (around 75 kDa) and truncated hFUS-Q290X (around 45 kDa) (*left* and *right* *columns*, respectively) under the control of *GMR*-*GAL4.* Note that although both protein extracts were run on the same gel, the blot were divided into two halves to be incubated with two different sources of the anti-FUS antibodies. This is due to the inability of both antibodies to cross react with the full length and the truncated hFUS. Note that the commercial antibody used to detect the full length protein does not detect the endogeneous dFUS in the control lane (*left* *panel*) as it recognises an epitope which maps to a region between residues 1 and 50 of hFUS, which is absent in dFUS (Stolow and Haynes 1995). The antibody used to detect the truncated hFUS protein was generated using a peptide at residues 130–140 of hFUS. Note that there is one strong band detected at the position expected for the truncated FUS protein. Currently, the presence of the lighter bands in the control lane (*right panel*) is still unclear, but those are likely to be unspecific signals. **c** Rescue of wing folded phenotype by knock down of endogenous *dFUS*. Ectopic expression of *hFUS*-*WT* with *Ddc*-*GAL4* results in wing folded phenotype (*middle panel*). This phenotype can be partially rescued by knocking down of endogenous *dFUS* expression (*right panel*), with either RNAi lines 32990 or 34839. Note that as compared with the control wings (*left panel*), rescued wings lack shiny appearance of the wild-type wing

To characterise the phenotypes associated with *hFUS*-*Q290X* mutation, we generated transgenic *Drosophila* carrying *hFUS* and targeted their expression in different tissues with the versatile binary *GAL4/UAS* system (Brand and Perrimon 1993). The effects of overexpression of *hFUS*-*WT* and *hFUS*-*Q290X* were analysed. As the *hFUS*-*Q290X* mutation has been previously shown to be degraded by the nonsense-mediated decay (NMD) pathway, we checked mRNA expression of *hFUS*-*WT* and *hFUS*-*Q290X* transgenes using RT-qPCR and found high levels of expression after 15 days of ageing (Fig. 1a). This was further confirmed by intact and similar expression levels of both the full length hFUS-WT and the truncated hFUS-Q290X proteins (shown on Western Blots, Fig. 1b). Overexpression of *hFUS*-*WT* in the dopaminergic and serotonergic systems resulted in the wing-folded phenotype which could be partially rescued by reducing the endogenous *dFUS* expression (Fig. 1c), suggesting conservation of genes and pathways between *Drosophila* and human.

**Fig. 2:**
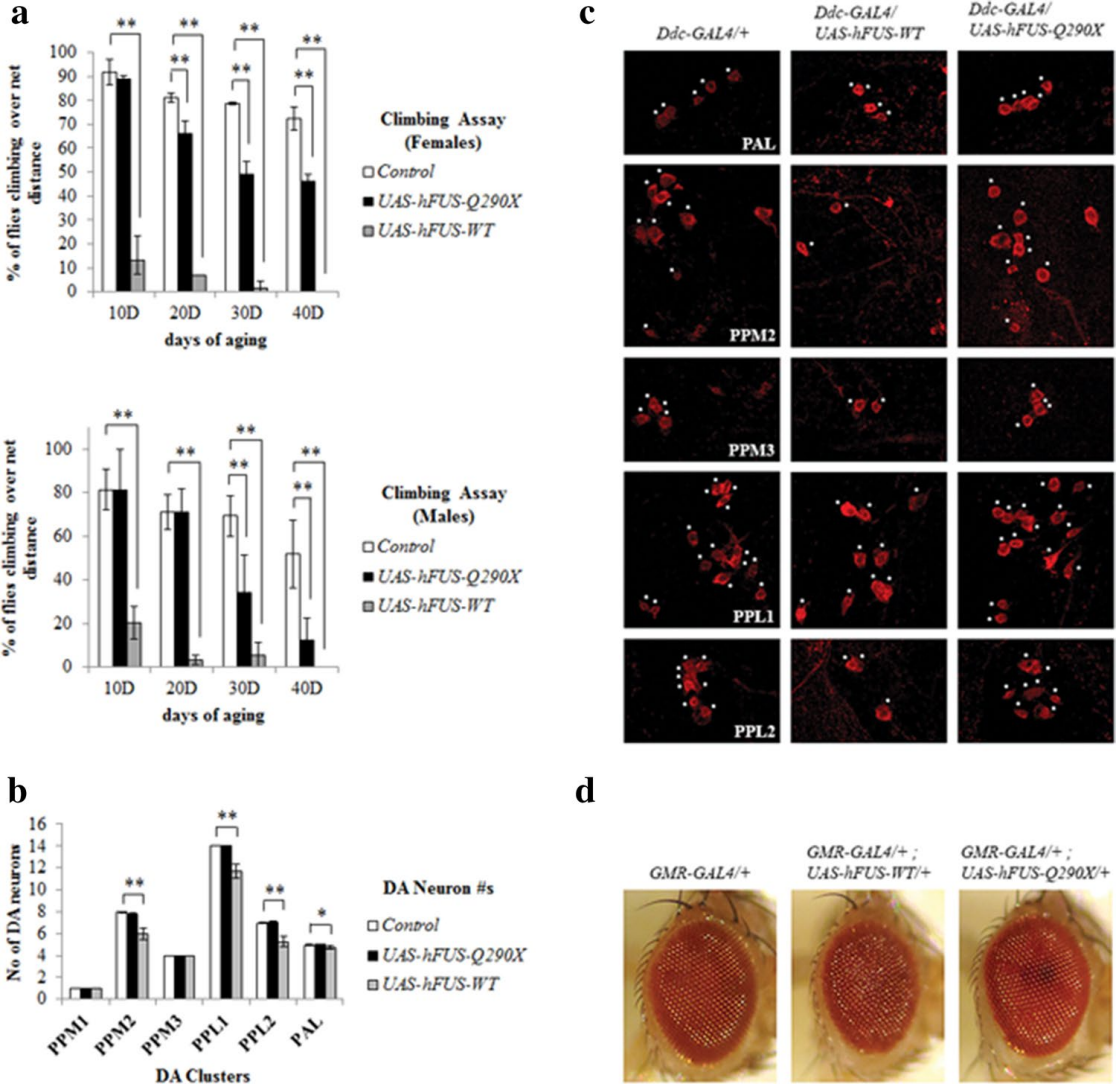
Targeted-expression of *hFUS*-*Q290X* results in movement impairment in the absence of neuronal degeneration. **a** Negative geotaxis assays (of females and males) reflecting locomotor ability of flies overexpressing *hFUS*-*Q290X* and *hFUS*-*WT* under the control of *Ddc*-*GAL4*. Control flies are *Ddc*-*GAL4/*+. Note that flies overexpressing *hFUS*-*Q290X* begins to show climbing impairment after 10 days of aging and this deteriorates rapidly after 30 and 40 days of aging. Overexpression of *hFUS*-*WT* starts to cause strong climbing impairment after 10 days of aging. Data shown are from three separate rounds of experiments (*n* = 3). Statistics used is Pearson Chi-square (*p* value ≤ 0.001 to indicate highly significance as denoted by **). **b** Quantification of DA neuronal clusters in the adult brain samples aged to 60 days (for *hFUS*-*Q290X* and *Control*) and to 40 days (for *hFUS*-*WT*). The quantification of DA neuron numbers in flies overexpressing *hFUS*-*WT* could only be carried out for samples aged till 40 days, because these flies did not survive to 60 days. Note that while reduction in neuronal numbers is significantly present in certain DA clusters of flies overexpressing *hFUS*-*WT*, there is no significant difference in the number of neurons between the control flies and the flies overexpressing *hFUS*-*Q290X*. Statistics used is one-way ANOVA with Bonferroni post-test for multiple comparisons (*p* value ≤0.001 to indicate highly significance as denoted by **; *p* value ≤0.05 to indicate significance as denoted by *). **c** Representative pictures showing DA neurons in the adult brain. The genotypes are: *Ddc*-*GAL4/*+ (control, *left column*); *Ddc*-*GAL4/UAS*-*hFUS*-*WT* (*middle* *column*); and *Ddc*-*GAL4/UAS* *-hFUS*-*Q290X* (*right* *column*). With the exception of the *Ddc*-*GAL4/UAS*-*hFUS*-*WT* samples which have been aged to 40 days, both the *Ddc*-*GAL4/*+ and the *Ddc*-*GAL4/UAS*-*hFUS*-*Q290X* samples have been aged to 60 days. Overexpression of *hFUS*-*WT* results in reduction of DA neurons; however, the number of DA neurons remains normal in flies overexpressing *hFUS*-*Q290X*. The main clusters of DA neurons examined are PAL (paired anterior lateral), PPM1, 2, 3 (paired posterior medial clusters 1, 2, and 3), PPL1, 2 (paired posterior lateral clusters 1 and 2). **d** Comparison of external structures of the adult ommatidia. Note that while overexpression of *UAS*-*hFUS*-*WT* (*middle panel*) results in mild rough eye, overexpression of *hFUS*-*Q290X* (*right panel*) causes no sign of degeneration as reflected by wild-type ommatidia similar to that seen in the control (*GMR*-*GAL4/*+, *left panel*)

Next, we wanted to analyze motor function in flies expressing these transgenes. Although, currently, we do not have an assay to measure tremor, we analysed locomotion using negative geotaxis (climbing) assay. As targeting expression of other neurodegenerative mutants in the dopaminergic and the serotonergic neurons using *Ddc*-*GAL4* has previously implicated those genes in motility (Angeles et al. 2014), we drove our transgenes with this driver. Overexpression of *hFUS*-*Q290X* resulted in progressive locomotor impairment (Fig. 2a, shown separately for females and males), suggesting a dominant-negative mode of action of the *Q290X* mutation. The observed locomotor impairment was not accompanied by obvious neuronal degeneration as shown by intact DA neurons when *hFUS*-*Q290X* was expressed with *Ddc*-*GAL4* (Fig. 2b, c). Since the presence of degeneration in the visual system can be reflected by external rough eye morphology, we expressed *hFUS*-*Q290X* in the visual system with *GMR*-*GAL4* and found wild-type eye morphology (Fig. 2d), suggesting absence of obvious degeneration. Impairments in climbing ability and degeneration were observed in flies overexpressing *hFUS*-*WT* (Fig. 2a–d), consistent with the previous reports (Chen et al. 2011; Mitchell et al. 2013).

To demonstrate that the observed phenotypes in flies overexpressing *hFUS*- *Q290X* were not due to toxicity as reported when *hFUS*-*WT* was overexpressed (Machamer et al. 2014), we drove expression of both transgenes with *OK371*-*GAL4*. We found that while overexpression of *hFUS*-*WT* resulted in pupal lethality, flies overexpressing *hFUS*-*Q290X* were viable to the adult stage without obvious abnormalities (data not shown).

### Expression of *hFUS*-*Q290X* results in impairement of the GABAergic system

**Fig. 3:**
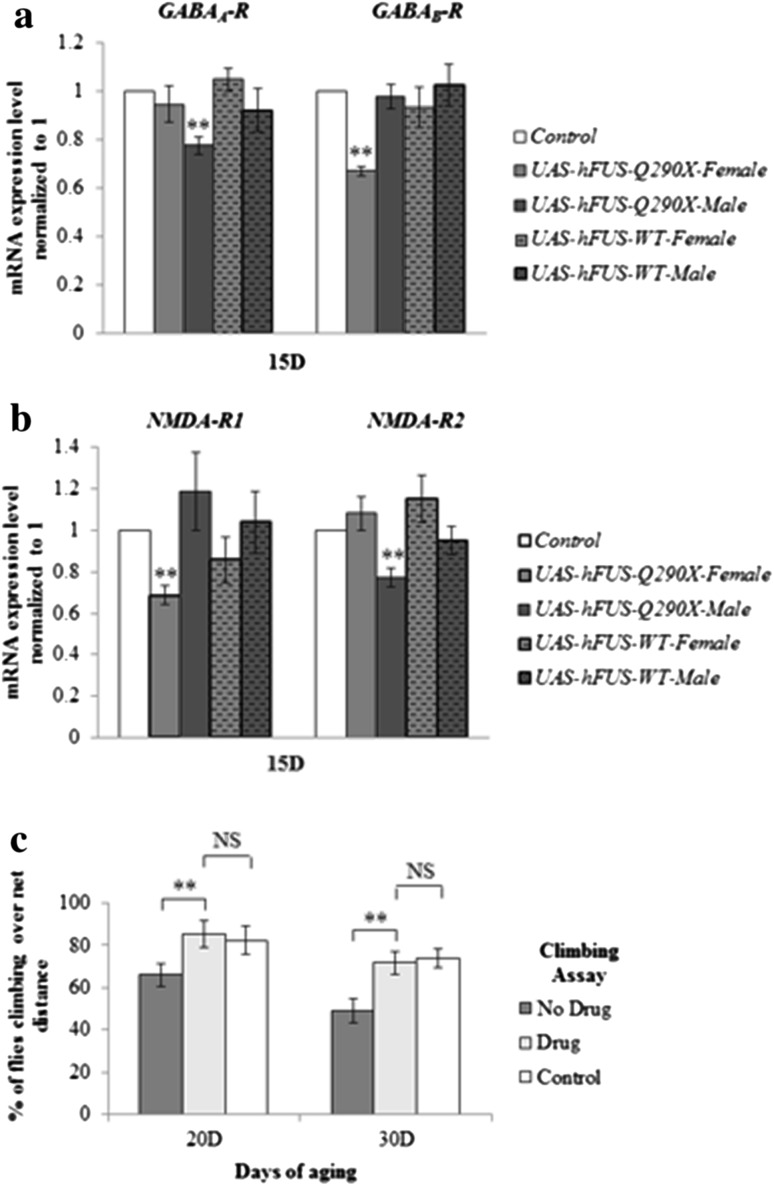
Expression of *hFUS*-*Q290X* causes impairment in the GABAergic system. **a**, **b** The mRNA expression levels of *GABA*-*R* and *NMDA*-*R* in flies overexpressing *hFUS*-*Q290X* and *hFUS*-*WT* under the control of *Ddc*-*GAL4*. Control flies are carrying one copy of *Ddc*-*GAL4* alone. All flies have been aged for 15 days before mRNA extraction. Results are normalised against the controls. Note that while *GABA*_*A*_-*R* is downregulated in the males, *GABA*_*B*_-*R* is downregulated in the females. Similarly, while *NMDA*-*R1* is preferentially downregulated in the females, *NMDA*-*R2* is preferentially downregulated in the males. The levels of *GABA*-*Rs* and *NMDA*-*Rs* are not significantly affected in flies overexpressing *hFUS*-*WT*. Statistical significance of RT-qPCR results is calculated using Student’s *t* test (*p* < 0.01, as denoted by **). **c** Rescue of climbing defect of female flies overexpressing *hFUS*-*Q290X* under the control of *Ddc*-*GAL4*. The ability of gabapentin to rescue the climbing defect is monitored for flies which have undergone 20 and 30 days of aging. Note that treatment with gabapentin is able to rescue the climbing ability of flies overexpressing *hFUS*-*Q290X* to very similar degree as those observed in the control flies. Statistical analysis was performed using Pearson Chi-square (with *p* values of 0.008 and 0.009 as denoted by ** for 20 days and 30 days of aging, respectively)

To determine the involvement of the GABAergic system in our overexpression study, we performed RT-qPCR analysis to check mRNA expression levels of the two major types of *GABA*-*R* in *Drosophila*, the ligand-gated ion channel-type *GABA*_*A*_-*R* and the metabotropic *GABA*_*B*_-*R* (Lei et al. 2013). As the GABAergic transmission has been shown to be regulated by N-methyl-d-aspartate (NMDA) receptor activation (Xue et al. 2011), we also analysed mRNA expression level of the two major *NMDA*-*R* subunits (*NMDA*-*R1* and *NMDA*-*R2*). Interestingly, not only that overexpression of *hFUS*-*Q290X* caused a decrease in the mRNA expression levels of these receptors (after aging to 15 days), but there also seemed to be gender specific differential downregulation of these receptor subunits (Fig. 3a, b). While *GABA*_*A*_-*R* was significantly downregulated in males, *GABA*_*B*_-*R* was significantly downregulated in females (Fig. 3a). Similarly, while *NMDA*-*R1* was preferentially downregulated in females, *NMDA*-*R2* was preferentially downregulated in males (Fig. 3b). It is interesting to note that although overexpression of *hFUS*-*WT* caused a strong locomotion phenotype, it did not significantly affect the expression levels of these receptors (Fig. 3a, b). This suggests that the observed locomotion phenotypes in flies overexpressing *hFUS*-*Q290X* and *hFUS*-*WT* were differentially regulated at the molecular level.

To further demonstrate that the impaired GABAergic system contributed to the decline in locomotion, we performed rescue experiment with gabapentin. Gabapentin was initially synthesised to mimic GABA and able to increase GABA biosynthesis and non-synaptic GABA neurotransmission (Kwan et al. 2001), and it is currently used in the clinic as one of the treatment options for ET (Deuschl et al. 2011). We found that gabapentin was able to rescue the locomotion phenotype caused by overexpression of *hFUS*-*Q290X* (Fig. 3c).

### Expression of *hFUS*-*Q290X* results in life span extension, downregulation of the insulin/insulin-like growth factor signalling (IIS) pathway and increase in serotonin expression

**Fig. 4:**
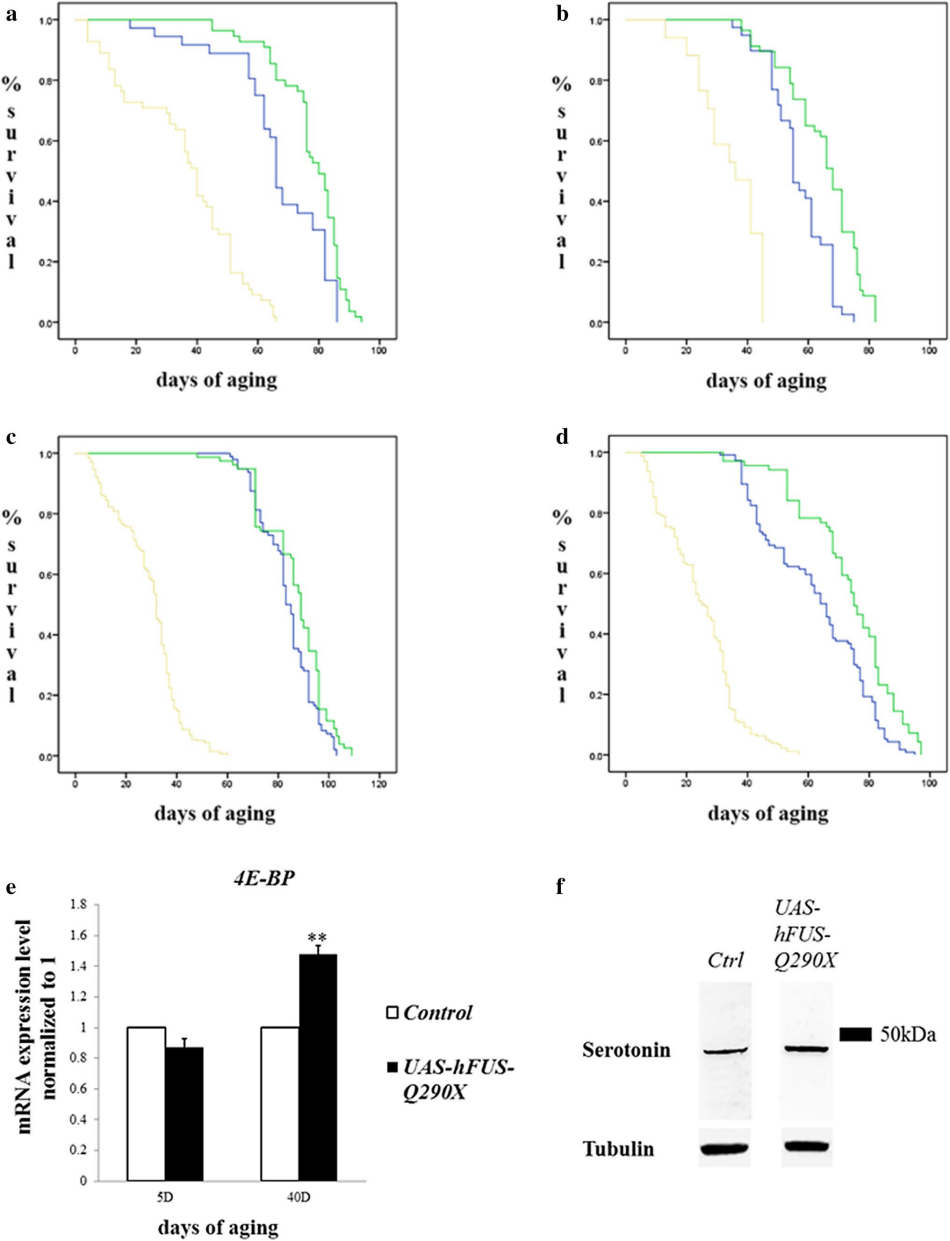
Expression of *hFUS*-*Q290X* causes increased longevity and downregulation of the IIS signalling pathway. **a–d** Survival curves (females and males shown separately) comparing longevity of control flies (*blue* in **a** and **b**, *ple*-*GAL4/*+ or **c** and **d**, *Ddc*-GAL4/+) vs. flies overexpressing one copy of *UAS*-*hFUS*-*Q290X* (*green*) or *UAS*-*hFUS*–*WT* (*yellow*). Note that while overexpression of *hFUS*–*WT* by either driver results in drastic mortality rate (*p* value ≤0.001), expression of *hFUS*-*Q290X* (**a** *p* value = 0.004; **b**, *p* value ≤0.001; **c** *p* value = 0.015; **d** *p* value ≤0.001) causes a significant increase in life span as compared with those of controls. The effect on life span becomes clearer when flies reached 50–70 days of age. Statistics are performed using Kaplan–Meier analysis with Cox regression. **e** RT-qPCR results comparing mRNA levels of *4E*-*BP* in control flies vs. flies overexpressing *hFUS*-*Q290X* under the control of *Ddc*-*GAL4* before aging (5 days post eclosion) and after aging (40 days post eclosion). Results are normalised against the controls. Note the increase in *4E*-*BP* mRNA expression level after 40 days of aging. Statistical significance of RT-qPCR results is calculated using Student’s *t* test (*p* = 0.008). **f** Western *Blotting* comparing serotonin level in control flies vs. flies overexpressing *hFUS*-*Q290X* under the control of *Ddc*-*GAL4*, which has been aged to 15 days. Note the increase in serotonin level in flies overexpressing *hFUS*-*Q290X* as compared with the control. Band quantification is carried out using Image J program, and statistical significance is performed using Student’s *t* test (*p* ≤ 0.001). In *all panels*, controls are flies carrying one copy of *Ddc*-*GAL4* alone

Next, we monitored longevity in our transgenic flies and found that while flies overexpressing *hFUS*-*WT* had short life span (yellow in Fig. 4a–d), flies overexpressing *hFUS*-*Q290X* lived significantly longer. The increase in life span was observed when *hFUS*-*Q290X* was overexpressed in the dopaminergic neurons (green in Fig. 4a, b) and in both the dopaminergic and the serotonergic neurons (green in Fig. 4c, d). As the insulin/insulin-like growth factor-like signalling (IIS) pathway has been implicated in the regulation of life span (Jia et al. 2004), we analysed the expression of one of the members (4E-BP) in this pathway. Our results showed that while *4E*-*BP* mRNA levels were not significantly different in young (5 days old) control flies and flies overexpressing *hFUS*-*Q290X*, its mRNA level was significantly increased in aged flies (40 days) (Fig. 4e), indicating downregulation of the IIS signalling pathway.

Serotonin has been known for its roles in controlling the GABAergic (Saitow et al. 2009) and the insulin (Luo et al. 2012) pathways. Using Western Blot analysis, we determined the level of serotonin and found that overexpression of *hFUS*-*Q290X* with *Ddc*-*GAL4* resulted in significant increase in the serotonin level in female flies (aged 15 days, Fig. 4f). Interestingly, the antibody that we used did not detect the presence of serotonin in male flies of same age. A likely explanation for this is that the antibody we used only recognised gender specific form of serotonin. Further experiments with different antibodies to test for gender specificity of serotonin changes would be helpful.

## Discussions

In this study, we found that despite previous report showing that the *hFUS*-*Q290X* truncation mutation was degraded by the NMD pathway, we could get stable expression of this transcript when expressed in *Drosophila*. It is likely that in ET patients the NMD pathway does not fully suppress the residual and possibly deleterious dominant-negative effect of molecules harbouring premature termination codons (PTCs) such as *hFUS*-*Q290X* and, hence, resulting in disease characteristics. The ability to express the truncated FUS protein enabled us to study its dominant-negative effect in *Drosophila* and revealed some insights on ET disease pathogenesis.

Our results show that flies overexpressing *hFUS*-*Q290X* had locomotion impairment in the absence of obvious degeneration. The locomotion impairment was accompanied by downregulation of neurotransmitter expression and the ability of rescue by gabapentin. These suggest that the *hFUS* gene may not be required for the specification or the maintenance of neurons but may be important at the level of neurotransmission. It is important to note that decreased levels of *GABA*-*R* had been observed in postmortem samples from ET individuals (Paris-Robidas et al. 2012). The observed gender specific effects on downregulation of the *GABA*-*R* and *NMDA*-*R* subunits expression also suggest the possible presence of sexual dimorphism in ET disease mechanisms. Sexual dimorphism occurs in both human (Morrow 2015) and *Drosophila*. In *Drosophila*, the sex difference in ethanol sedation has been linked to the expression of a gene that regulates splicing of *fruitless* (*fru*) (Devineni and Heberlein 2012). Male specific Fru^M^ isoforms exist which regulate sex-specific neural circuits responsible for regulating courtship behaviour (Von Philipsborn et al. 2014). Due to the high conservation of genes and genetic pathways in both human and flies, it is possible that levels of serotonin, *GABA*-*R* and *NMDA*-*R* showed gender specific differences in our experimental paradigm. It will be interesting to evaluate gender specific differences in clinical drug studies as this can provide an explanation for inefficient drug therapy or adverse effects which may be caused by overactivation of certain pathways in some individuals.

Increased longevity has been associated with cases of early onset ET. However, the opposite was noted in patients having late onset ET, possibly complicated by other comorbidities. It is to be noted that the *hFUS*-*Q290X* mutation was identified in patients having early onset ET, and our data show that overexpression of *hFUS*-*Q290X* resulted in prolonged life span which was accompanied by downregulation of the IIS/TOR signalling pathway. One of the downstream molecules in the IIS/TOR pathway is Forkhead bOX-containing protein, subfamily O (FOXO) (Kahn 2015), and a direct target of FOXO is 4E-BP. Interestingly, *FOXO3A* genotype has also been strongly associated with longevity in human (Willcox et al. 2008).

Since serotonin is known for its role in controlling the GABAergic and the insulin pathways, it is likely that upstream of all the observed phenotypes in our overexpression analysis is caused by impairment in the serotonergic pathways. Our observation of a possible role of serotonin on the control of locomotion is corroborated by studies linking increased extracellular serotonin level to impaired larval locomotion through application of drugs that block serotonin transporter (Dasari et al. 2007). Further studies will be required to decipher how the GABA receptors, and the different serotonin receptors or combinations of receptors interact and regulate the resultant receptor signalling in FUS-linked ET.

In summary, we demonstrated that *Drosophila* expressing *hFUS*-*Q290X* could recapitulate certain characteristics found in ET patients such as increased motor dysfunction with age, absence of obvious neuronal loss and possibly longer life span. These phenotypes were accompanied by gender specific reduction in the expression of *GABA*-*R* and *NMDA*-*R* subunits, rescue of the motor dysfunction with gabapentin, downregulation of the IIS/TOR signalling pathway and increase in serotonin level. Hence, our studies of *hFUS*-*Q290X* in in vivo *Drosophila* model have shed some mechanistic insights on ET pathogenesis. Further phenotypic characterization and evaluation of the involved molecular mechanisms may lead to improvement of clinical diagnosis and therapy for ET.
